# The importance of the quaternary structure to represent conformational ensembles of the major *Mycobacterium tuberculosis* drug target

**DOI:** 10.1038/s41598-019-50213-0

**Published:** 2019-09-23

**Authors:** Renata Fioravanti Tarabini, Luís Fernando Saraiva Macedo Timmers, Carlos Eduardo Sequeiros-Borja, Osmar Norberto de Souza

**Affiliations:** 1 Laboratório de Bioinformática, Modelagem e Simulação de Biossistemas (LABIO), Pontifícia Universidade Católica do Rio Grande do Sul (PUCRS), Av. Ipiranga 6681, 90619-900 Porto Alegre, RS Brazil; 2 Programa de Pós-Graduação em Biologia Celular e Molecular, PUCRS, Porto Alegre, RS Brazil; 3 Present Address: Programa de Pós-Graduação em Biotecnologia (PPGBiotec), Universidade do Vale do Taquari -Univates, Rua Avelino Talini, 171 - Bairro Universitário, Lajeado, RS Brazil; 4 Present Address: Faculty of Biology, Institute of Molecular Biology and Biotechnology, Department of Gene Expression, Laboratory of Biomolecular Interactions and Transport, Adam Mickiewicz University in Poznań, Poznań, Poland

**Keywords:** Molecular modelling, Computational biophysics

## Abstract

Flexibility is a feature intimately related to protein function, since conformational changes can be used to describe environmental changes, chemical modifications, protein-protein and protein-ligand interactions. In this study, we have investigated the influence of the quaternary structure of 2-*trans*-enoyl-ACP (CoA) reductase or InhA, from *Mycobacterium tuberculosis*, to its flexibility. We carried out classical molecular dynamics simulations using monomeric and tetrameric forms to elucidate the enzyme’s flexibility. Overall, we observed statistically significant differences between conformational ensembles of tertiary and quaternary structures. In addition, the enzyme’s binding site is the most affected region, reinforcing the importance of the quaternary structure to evaluate the binding affinity of small molecules, as well as the effect of single point mutations to InhA protein dynamics.

## Introduction

Flexibility is a feature intimately related to protein function since conformational changes in protein structures can be used to describe environmental changes, chemical modifications, in addition to protein-protein and protein-ligand interactions^1–3^. Several research groups have sought to demonstrate the importance of protein flexibility at different levels. Weinkam and coworkers^4^ described mutations in a protein sequence that could be associated with changes in the allosteric conformational equilibrium. Plattner and Noé addressed the importance of conformational plasticity in receptors based on molecular dynamics simulations (MD) and Markov state models^5^. McCammon and coworkers^6^ also used MD simulations to provide insights into the function and mechanism of RNA polymerase II reactivation by the transcription factor IIS. In another work, Amaral and collaborators^7^ highlighted the importance of protein flexibility in structure-based drug discovery projects, and more recently, Bringas and coworkers^8^ showed the importance of tertiary and quaternary structures’ flexibility to regulate oxygen affinity in human hemoglobin based on classical and hybrid MD simulations. Altogether, these works provide an overview of how the knowledge on protein flexibility achieved through computer simulations can help to explain biological phenomena.

InhA or 2-*trans*-enoyl-ACP (CoA) reductase (EC 1.3.1.9) from *Mycobacterium tuberculosis* (Mt) is among the most widely studied enzymes for being the target of the first-line antitubercular drug isoniazid^9^. The enzyme MtInhA (InhA from Mt) has been investigated through different computational approaches to describe how inhibitor candidates bind to the active site^10–12^, the hot-spot residues responsible for inhibitor binding^13^, as well as how point mutations, such as I21V, trigger isoniazid resistance^14^. This latter study was immediately confirmed using experimental validation^15^. Still, another work by Tonge, Simmerling and coworkers explored conformational changes in MtInhA to explain experimental data on the modulation of slow-onset inhibitors^16^. Furthermore, MD simulations showed that the association of ligands in the MtInhA substrate-binding pocket (SBP) could directly modulate the flexibility of its substrate-binding loop (SBL), A-loop, and B-loop^10,13^. Schroeder and coworkers^14^ showed considerable flexibility of the SBL motif by using the monomeric form of the MtInhA. Despite all computational studies on the MtInhA flexibility, the impact of the quaternary structure was not taken into account since all information was obtained from the tertiary structure simulation only. The use of the monomeric MtInhA form in many computational studies has been supported by the fact that its binding sites occur about 40 Å apart from each other^14,17^, suggesting that they are independent of each other. However, according to the tetrameric organization, the binding cavity is close to the monomers interface and two of the major motifs of the substrate-binding pocket (SBP), the A- and B-loops. It interacts directly with the adjacent subunit, suggesting that the quaternary structure could be involved in the modulation of the MtInhA flexibility. Besides, a detailed structural analysis of all available MtInhA crystal structures corroborated the hypothesis in which the A- and B-loops have lower values of B-factors in relation to the SBL, which faces the outside of the tetramer toward the solvent^18–21^.

This work consists of an extensive MD simulation study to evaluate the importance of the quaternary structure to the MtInhA flexibility by simulating three MtInhA systems: (i) the apo form, (ii) bound to NADH, and (iii) bound to NADH and a substrate analog. Seeking to understand the role of the quaternary structure in the modulation of the MtInhA flexibility, the tertiary and quaternary structures were simulated for all systems. We subsequently applied probability density functions and principal component analysis to assess the conformational ensembles obtained from the MD simulations in addition to using an ANOVA analysis to describe the significance of the observed differences between the means of the simulated systems. Comparisons of the flexibility between the tertiary and quaternary structures led to relevant insights into the conformational changes undertaken by this enzyme.

## Material and Methods

### MtInha crystal structures

We used three different systems to evaluate the impact of the quaternary structure on the MtInhA flexibility: (i) the apo form, (ii) bound to NADH, and (iii) bound to NADH and substrate analog (THT). All systems were considered at two levels of structural organization: the tertiary (monomer) and quaternary (tetramer) forms. Figure SF1 shows the arrangement of the subunits in the tetrameric and monomeric forms used in all simulations. All crystal structures were obtained from the Protein Data Bank (PDB). Since the crystal structure of the MtInhA apo form lacks the helix H6 of the SBL, we decided to use the PDB entry 1ENY without the NADH molecule as our starting structure for the apo form. For the systems involving binary (associated with the NADH molecule) and tertiary complexes (bound to NADH:THT), we employed the PDB entries 1ENY and 1BVR, respectively^20,22^. Finally, we used the rotational and translational matrices deposited in the PDB to generate the quaternary structures.

### Molecular mechanics and dynamics protocol

The molecular mechanics (MM) and MD simulations of the MtInhA enzyme were carried out using the Amber99SB force field^23^ implemented in GROMACS 2016.3^24^. Periodic bound conditions were applied and the numbers of particles, pressure, and temperature were maintained constant (NPT ensemble) during the whole production phase. The periodic boundary boxes of monomeric and tetrameric MtInhA systems were composed of 15,116 and 47,449 water molecules, respectively. The V-rescale^25^ thermostat was employed to maintain the system at constant temperature using a coupling time of 0.1 ps, and the Berendsen barostat^26^ was applied to ensure that the system pressure was maintained at 1 bar. The LINCS algorithm^27^ was implemented to constrain all of the covalent bonds involving hydrogen atoms so that the systems could evolve in a time step of 2 fs. van der Waals interactions were computed using a 12.0 Å cutoff. The particle mesh Ewald (PME) method was used to calculate electrostatic contributions in a grid with 1.2 Å spacing. The macromolecule was fully solvated using the TIP3P model^28^ in a cubic box extending 10.0 Å from the macromolecule surfaces. The systems were submitted to steepest-descent energy minimization up to a tolerance of 1,000 kJ mol^−1^ nm^−1^ in order to remove close contacts of van der Waals forces. The equilibration phase was performed in two steps: (i) a NVT equilibration for 500 ps followed by (ii) a NPT equilibration step lasting 1,000 ps before the production phase. We performed three different simulations for each MtInhA system by using different random seeds. The production phase of each MD simulation lasted for 100 ns and the total simulation time was 1.8 μs. All analyses were carried out using the last 90 ns of each simulation.

### Structural analysis of the MtInhA enzyme

**Figure 1:**
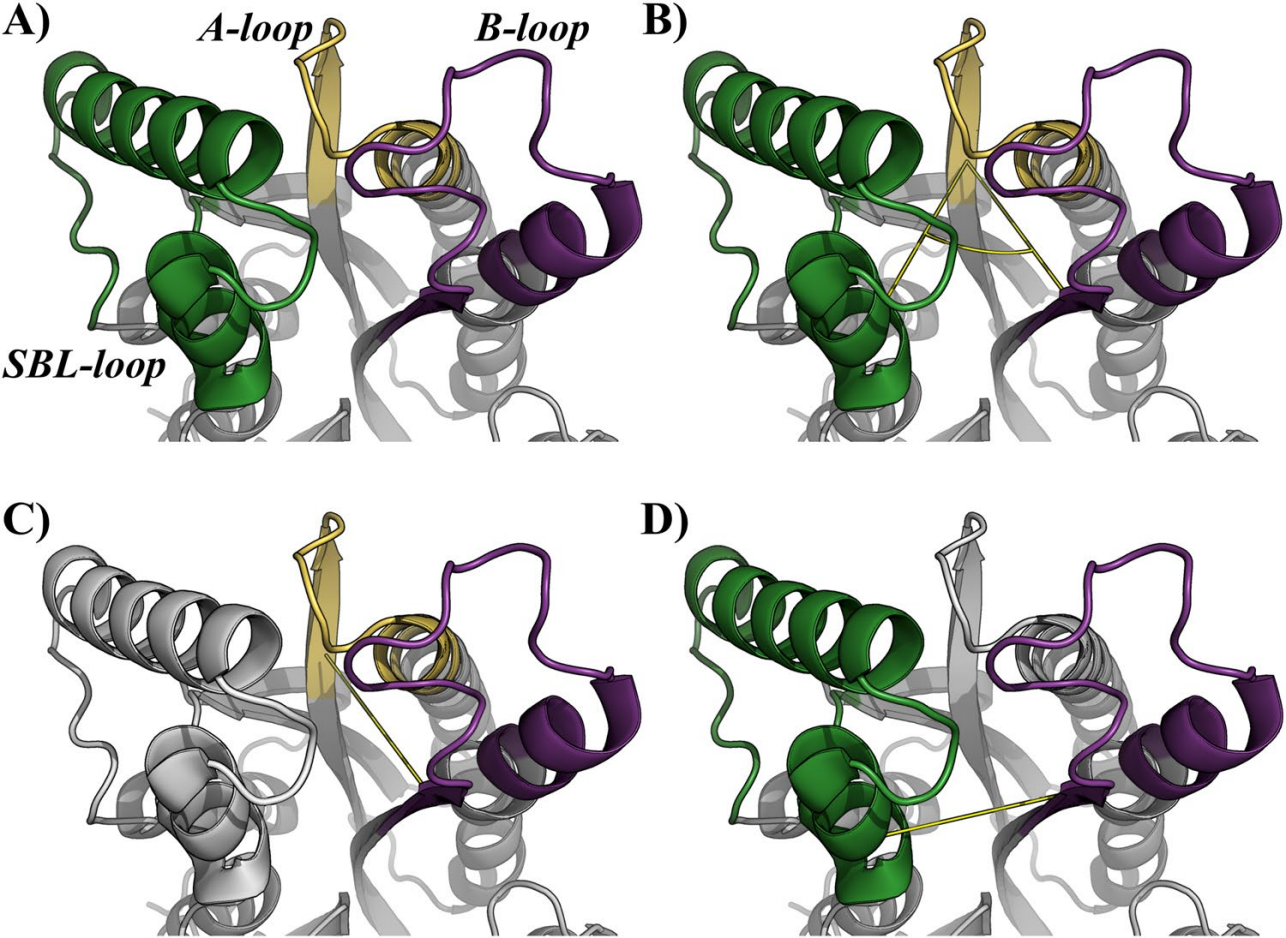
Definition of the structural analysis. The substrate binding cavity is defined by three motifs, the SB-loop (green), A-loop (purple) and B-loop (yellow). (**A)** Description of the motifs involved in the analyses. (**B)** Definition of the pincer angle and triangle area. (**C)** A-loop and B-loop distance, and (**D)** SB-loop and A-loop distance. The main chain of the enzyme is represented as *cartoon*. Image generated with PyMOL^36^.

To describe the impact of the quaternary structure in the MtInhA flexibility, the conformational ensembles were analyzed according to the distances, angle, and area variations defined in the SBP. All analyses were performed based on the three motifs of the SBP, (i) A-loop (residues F96, M97, P98, Q99, T100, G101, M102, G103, I104, N105, P106, F107, F108, D109, A110, P111,Y112, A113, D114, V115, S116, K117, G118, I119, H120), (ii) B-loop (residues D149, P150, S151, R152, A153, M154, P155, A156, Y157, N158, W159, M160, T161, V162, A163, K164, S165, A166), and (iii) SBL (residues P192, I193, R194, T195, L196, A197, M198, S199, A200, I201, V202, G203, G204, A205, L206, G207, E208, E209, A210, G211, A212, Q213, I214, Q215, L216, L217, E218, E219, G220, W221, D222, Q223, R224, A225, P226, I227, G228, W229, N230, M231, K232, D233). To monitor the course of SBP open and closure, a pincer angle was defined (Fig. 1B) based on the mass center of the motifs A- and B-loops, and the SBL. Furthermore, the triangle area formed by the pincer angle was assessed to monitor the distances between the SBL and A-loop (Fig. 1D), as well as between the A- and B-loops (Fig. 1C) to gather further information of the influence of the quaternary structure on them. The root mean square deviation (RMSD) of the residues Y158 and K165 was assessed, considering their importance to the catalytic mechanism^29^. All analyses were carried out on GROMACS 2016.3 package with in house scripts.

### Principal component analysis

Nowadays, MD simulations produce huge amounts of data to describe protein function and dynamics, protein-ligand, and protein-protein interactions at microscopic details. However, it is important to reduce the dimensionality of these data sets to extract functionally relevant information. Aiming at better understanding the differences among the systems, a principal component analysis was used to analyze the internal collective motions of all systems simulated as a function of the Cα atoms by calculating a covariance matrix and its eigenvectors. The modules “gmx covar” and “gmx anaeig” from GROMACS were used to calculate the eigenvalues and eigenvectors of the MD trajectories and to obtain a description of the most flexible regions of each system, respectively. The assumption that the monomers in the tetramer have similar behaviors was taken. Therefore, the root mean square fluctuations (RMSF) for the tetrameric systems correspond to a mean of all subunits in order to establish a comparison between monomeric and tetrameric structures. The principal component analyses (PCA) were performed for all simulations (3 × 100 ns) and for each condition (apo MtInhA, MtInhA:NADH, and MtInhA:NADH-THT). All analyses were carried out using the last 90 ns of each simulation.

### Statistical analysis

Statistical analyses were performed on RStudio^30^. One-way analysis of variance (ANOVA) was used to evaluate the occurrence of statistically significant differences between the means of the systems simulated. Since Levene’s statistics test^31^ revealed an unequal variance of the system distributions, we applied the Games-Howell^32^ test at 95% of confidence. Bonferroni *post hoc* correction test was applied to ensure that relative false-positive rate applied to the data set does not exceed the specific value (p adjusted < 0.01). The null hypothesis (H0) was that the monomer structure has the same mean distribution as the tetramer monomers. The alternative hypothesis (HA) was that the mean distribution of the monomer structure was different from the monomers in the tetramer.

### Interaction energy analysis

In order to assess the interaction energy of the quaternary structure of MtInhA, each tetramer system was analyzed with the “AnalyseComplex” command of FoldX software v4.0^33^. All snapshots from the simulation were partitioned into separate PDB files, and for each of them, the interaction energy of all interfaces was measured, i.e. A-B, A-C, A-D, B-C, B-D and C-D interfaces.

## Results and Discussion

MM/MD simulations were performed to gain insight and describe the impact of the quaternary structure on the flexibility of the MtInhA enzyme. Specifically, the comparisons allowed highlighting the influence of the tetrameric structure in the flexibility of the A-loop, B-loop, and the SBL. Moreover, it was possible to demonstrate whether restrictions in the conformational changes of these motifs are due to tetramer contacts or not.

### Comparison of monomeric and tetrameric ensembles of the apo MtInhA

**Figure 2:**
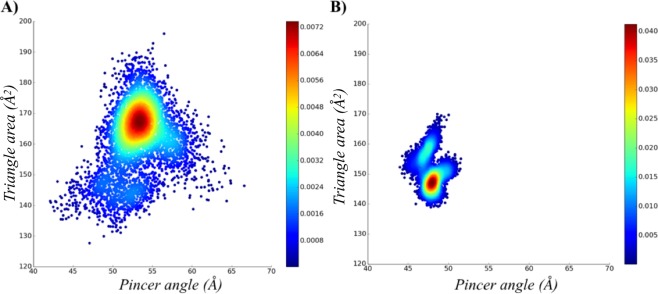
Description of the apo conformational ensembles of MtInhA. The probability density plots of (**A**) monomeric and (**B**) tetrameric forms highlight the difference between the conformational ensembles. Image generated with MatPlotlib^37^.

**Figure 3:**
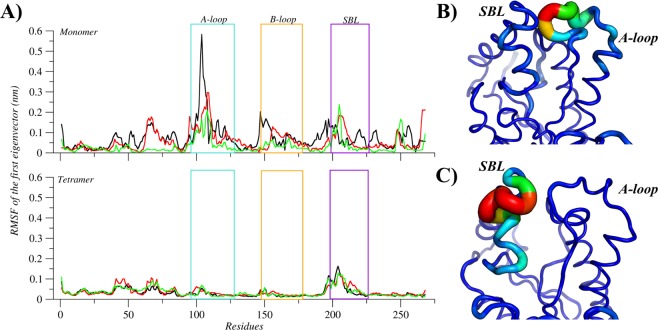
Flexibility of the apo MtInhA. (**A**) Root mean of the root mean square fluctuation (RMSF) for the monomeric and tetrameric systems, according to the first eigenvector. The regions A-loop, B-loo, and SBL are highlighted with boxes and coloured in light blue, orange, and purple, respectively. In addition, black, red and green lines represent the three simulations’ repetitions. The mobility and conformation change of the most representative structure extracted from the MD trajectories for monomer (**B**) and tetramer (**C**). The thickness is proportional to B-factor values. Image generated with XMGrace^38^ and PyMOL^36^.

The analysis of the relationship between area and pincer angle shows that the simulations of the apo structure were able to explore the conformational space considering the absence of interactions with small molecules in the active site. However, by comparing the tertiary and quaternary structures, the monomeric system of apo MtInhA proved to sample conformations which are not accessible to the tetrameric system; both systems have substantially different values for the most populated conformation. Figure 2 illustrates the conformational ensembles of the tertiary and quaternary structures according to the probability density function. The monomeric system has a more spread plot, with the triangle area and the pincer angle of the most representative conformation averaging 167.6 Å^2^ and 51.4°, respectively, whereas for tetrameric system conformation the values are 148.8 Å^2^ and 47.4°, respectively. Overall, the results suggest that the volume of the substrate-binding cavity increases because the tertiary structure has no adjacent subunits to make contacts. The increased volume observed for monomeric structures is due to higher flexibilities of the A-loop, B-loop, and SBL. Moreover, the RMSF of the first eigenvector, reveals three flexible motifs (A-loop, B-loop, and SBL) in the monomeric form, whilst the tetrameric system has only SBL as a flexible motif (Fig. 3). PCA analysis also demonstrated that the total positional fluctuations described by the first 50 eigenvectors are 91.63%, 56.18%, and 85.78% for the monomer simulations and 73.33%, 76.95%, and 77.02% (Table ST1) for the tetrameric simulations.

The representative conformation of the tetramer ensemble has higher flexibility in the SBL, whereas the A- and B-loops are more rigid than in the monomeric ensemble. The rigidity of A- and B-loops results from protein-protein contacts between the adjacent subunits in the tetramer’s quaternary structure. Analyses of the means from the monomeric and tetrameric distributions revealed statistically significant differences (p-value < 0.01). The distances between the A- and B-loops have pronounced differences when comparing monomeric and tetrameric systems, with the most representative conformations averaging 16.5 Å and 21.3 Å in the monomeric and tetrameric systems, respectively. Additionally, comparisons of the distance between the SBL and A-loop, which averages to18.8 Å and 22.0 Å, respectively, further corroborates the distinctive MtInhA dynamics in the monomeric and tetrameric systems. The visual differences were confirmed through statistical analysis yielding p-values of < 0.01 for all comparisons between apo monomeric and tetrameric forms, indicating a statistically significant difference between their distributions (Table ST2–ST5).

### Comparison of monomeric and tetrameric ensembles of the MtInhA holo form

**Figure 4:**
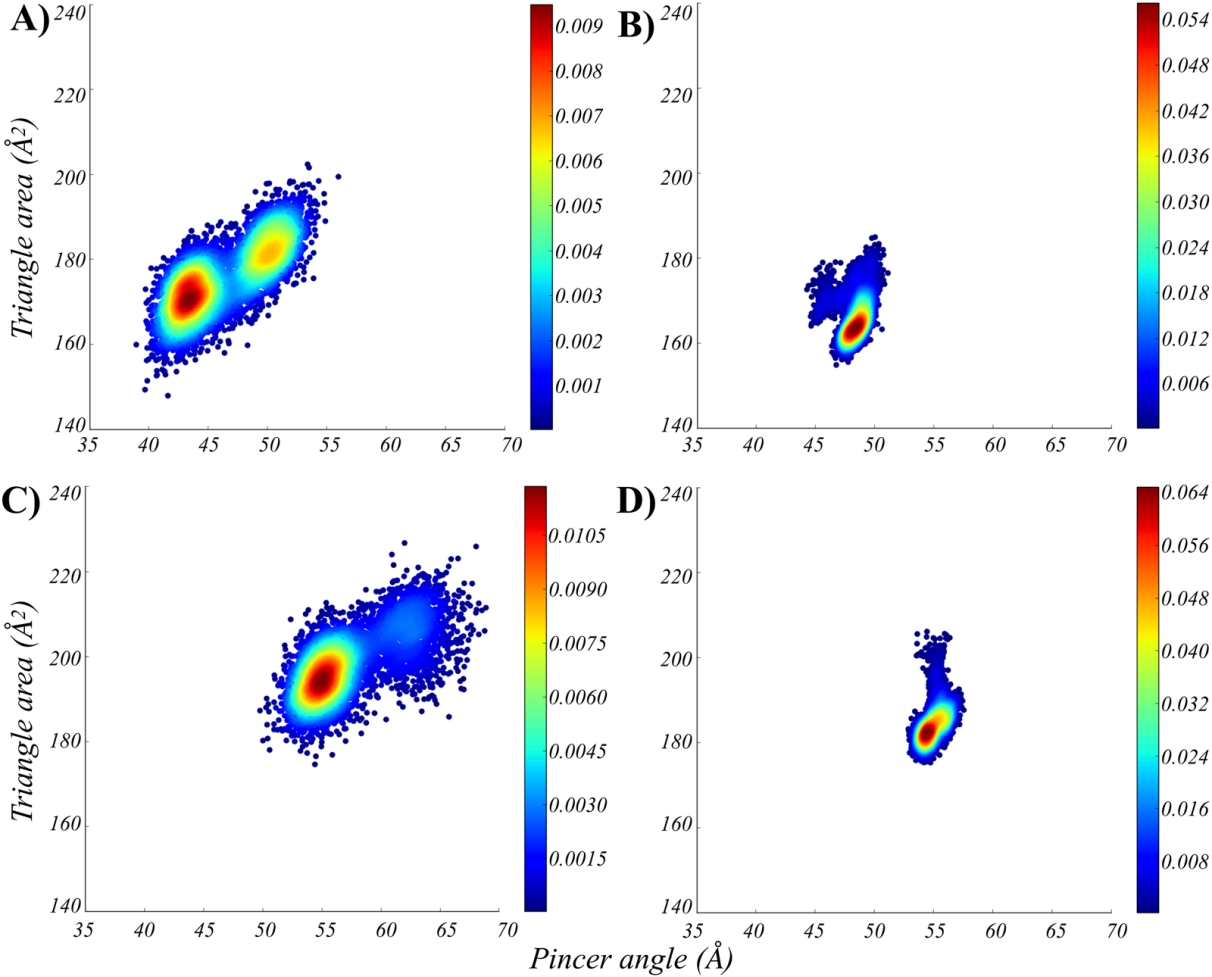
Description of the conformational ensembles of MtInhA associated with NADH and NADH:THT. The probability density plots of (**A**) monomeric MtInhA:NADH, (**B**) tetrameric MtInhA:NADH, (**C**) monomeric MtInhA:NADH:THT, and (**D**) tetrameric MtInhA:NADH:THT highlights the differences of the conformational ensembles. Image generated with MatPlotlib^37^.

**Figure 5:**
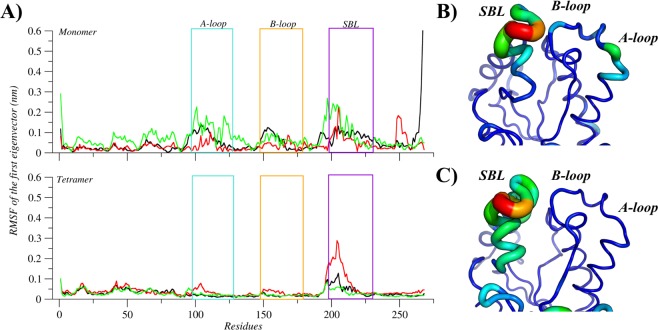
Flexibility of the MtInhA associated with NADH. (**A**) Root mean of the root mean square fluctuation (RMSF) for the monomeric and tetrameric systems, according to the first eigenvector. The regions A-loop, B-loo, and SBL are highlighted with boxes and coloured in light blue, orange, and purple, respectively. In addition, black, red and green lines represent the three simulations’ repetitions. The mobility and conformation change of the most representative structure extracted from the MD trajectories for monomer (**B**) and tetramer (**C**). The thickness is proportional to B-factor values. Image generated with XMGrace^38^ and PyMOL^36^.

**Figure 6:**
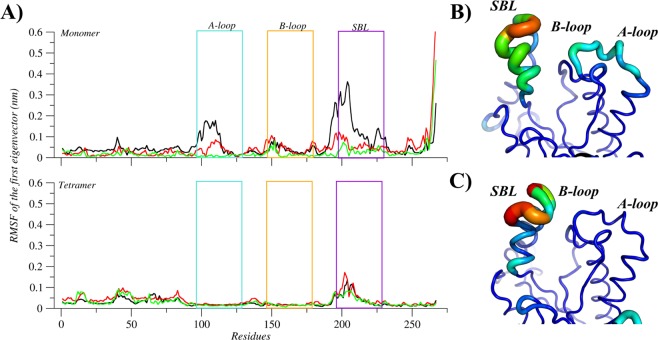
Flexibility of the MtInhA associated with NADH and substrate analogue (THT). (**A**) Root mean of the root mean square fluctuation (RMSF) for the monomeric and tetrameric systems, according to the first eigenvector. The regions A-loop, B-loo, and SBL are highlighted with boxes and coloured in light blue, orange, and purple, respectively. In addition, black, red and green lines represent the three simulations’ repetitions. The mobility and conformation change of the most representative structure extracted from the MD trajectories for monomer (**B**) and tetramer (**C**). The thickness is proportional to B-factor values. Image generated with XMGrace^38^ and PyMOL^36^.

The dynamic behavior of the MtInhA holo form is also affected by the presence of the quaternary structure, mainly on the binding pocket. MtInhA monomeric and tetrameric ensembles associated with NADH and NADH:THT shows substantial differences regarding the conformational space sampled. Figure 4 shows the probability density plots of monomeric and tetrameric ensembles of MtInhA:NADH (Fig. 4A,B) and MtInhA:NADH:THT (Fig. 4C,D). The most representative conformation of the monomeric MtInhA:NADH system presents a more compact conformation for the substrate-binding cavity with a pincer angle of 42.1° and an area of 171.0 Å^2^. It is worth emphasizing that the value obtained for the pincer angle in the monomeric system does not occur in the tetrameric ensemble, in which the pincer angle of the most representative conformation is 48.0° with an area of 162.0 Å^2^. Such a difference could also be associated with the quaternary structure since the monomeric system has no adjacent subunits to restrict its flexibility. The monomeric ensemble can access conformations regarding the triangle area parameter, which is also described by the tetrameric ensemble; however, these conformations represent only transient states and are not local minimum structures. It is important to note that only the quaternary structure can stabilize the region Ser152-X6-Tyr158-X6-Lys165, which contain the signature of short-chain dehydrogenase family of protein, and is essential to substrate recognition and enzyme catalysis. Furthermore, the analysis of the distances between the SBL and A-loop, as well as between the A- and B-loop, also shows a significant difference in the conformational space sampled by the binary and ternary complexes in monomeric and tetrameric forms (Fig. SF2). Our statistical analysis supports the difference between the distributions of monomeric and tetrameric ensembles of the MtInhA:NADH, yielding p-values of <0.01 (Tables ST1–ST4). The ternary complex (MtInhA:NADH:THT) also behaves similarly to the apo form and the binary complex (MtInhA:NADH) (Fig. 4C,D), highlighting the differences in protein dynamics in monomeric and tetrameric ensembles. Figures 5 and 6 emphasize the regions involved in an increased flexibility in relation to tertiary and quaternary structure. It is important to observe an intrinsic relation of A-loop, B-loop, and SBL to the dynamics behavior of the MtInhA binding pocket. All PCA analyses regarding the contributions of the eigenvectors are described in Table ST1.

The monomeric ensemble shows a more spread distribution regarding the pincer angle, and also statistical analysis shows that the means of monomeric and tetrameric distributions are statistically different (p-value < 0.01). Considering these results, the monomeric ensemble diverges from all tetrameric subunits, demonstrating the importance of the quaternary structure to study the MtInhA flexibility. These findings reinforce the importance of the quaternary structure to evaluate the binding affinity of small molecules, as well as to investigate the effect of single point mutations in protein dynamics.

### Importance of the tetrameric conformation for MtInhA

To explain the structural differences that define the flexibilities of the A-, B- and SBL loops in the monomeric and tetrameric conformations of MtInhA, two analyses were carried out: (i) an energetic analysis of the interface regions using the module *AnalyseComplex* from FoldX, and (ii) the RMSD for the A-, B- and SBL loops. From the energetic profiles of each interface (Fig. SF3), it can be observed that three groups are formed and maintained through all systems (apo MtInhA, MtInhA:NADH, MtInhA:NADH:THT). The interfaces A-D and B-C are the most stable, presenting an average energy of −132.0 kJ, followed by A-C and B-D interfaces (average −63.0 kJ), and the most unstable interactions were observed for the A-B and C-D interfaces (average −30.0 kJ). Therefore, interfaces A-D and B-C are the ones that bring more stability to the tetrameric form. In addition, it is important to mention that interface A-D is the specular image of interface B-C. In order to obtain more information about the importance of interfaces on the studied loops, the percentage of the loop’s residues belonging to each interface was quantified (Table ST6). From the results, it can be observed that the residues of the SBL loop take a small portion of the interfaces, presenting ≈10% (A-B and C-D) and ≈7% (A-C and B-D). This result is corroborated with RMSF values, where SBL loop has a higher flexibility when compared to A- and B-loops. B-loop is in contact with all subunits, and ≈66% of its residues take part in the interfaces A-D and B-C. In addition, A-loop takes part only in A-D and B-C interfaces, where 40% of its residues are mediating interactions. Figures SF4 and SF5 show the RMSD results for the monomeric and tetrameric conformations respectively. It can be observed, based on the RMSD, that the A-loop and B-loop are more flexible in the monomeric than in the tetrameric form. These results suggest a mechanism of regulation, exerted by the protein, for the flexibility of these regions. Interestingly, the dynamics of SBL-loop is similar in both monomeric and tetrameric forms. Altogether, these results suggest why the tetrameric form is essential to represent a reliable conformation ensemble better since its interfaces are limiting the flexibility of key binding pocket’s regions. It can be observed by comparing the RMSF results for all systems (Figs 3, 5 and 6) that the difference in flexibility between monomeric and tetrameric forms is evident for the A-loop. Taking into account the data obtained from the energetic and RMSD analysis, we identified three main findings: (i) A-loop is the one with major differences between monomeric and tetrameric forms, especially in the apo form. This behavior could be explained by the fact that half of A-loop’s amino acid residues takes part of the strongest interfaces (A-D and B-C), maintaining the loop’s rigidity in the tetrameric form. Hence, the A-loop exhibits artificial higher flexibility in the monomeric form, since interfaces are inexistent. (ii) The B-loop shows similar flexibility in monomeric and tetrameric forms (≈0.1 nm difference) even though it takes an active part in almost all interfaces, suggesting that the flexibility of this region is not directly modulated by the quaternary structure. The B-loop is located between SBL and the A-loop (Fig. 1); these loops may be limiting its flexibility. (iii) The SBL flexibility is very similar in the monomeric and tetrameric forms, since most of its residues does not mediate interactions at the interfaces of the tetramer. Moreover, unlike the B-loop, which is located between the A- and SBL loops, the SBL loop faces the solvent, allowing an unrestricted movement that could explain its higher flexibility.

## Conclusion

In previous works, the MtInhA enzyme has been used as a monomer in MM/MD simulations^9,34,35^, based on the assumption that the binding sites are independent of each other and thereby the tertiary structure could represent the behavior of the biological structure in solution. However, based on our results, there is a statistically significant difference between the conformational ensembles of tertiary and quaternary structures. Our analyses show that the MtInhA active region is much more flexible in the monomeric than the tetrameric form. Furthermore, by comparing the motions of A-loop, B-loop, and SBL, we observed that in the quaternary structure, flexibility is restricted to the SBL motif, while in the monomeric (tertiary structure), flexibility varies among all these motifs. These differences could be associated with the tetrameric form in contact with an adjacent subunit due to the quaternary packing. Moreover, statistical comparisons between the conformational ensembles of monomeric and tetrameric forms yielded p-values < 0.01, indicating a statistically significant difference between the means of these distributions. The MM/MD simulations allowed us to observe that the tetramer subunits can exhibit conformational ensembles without significant differences. These findings suggest that the use of the MtInhA quaternary structure may be mandatory in studies of its function and dynamics, as well as in virtual screening using multi-conformation or ensemble-docking approaches. Altogether, we believe these results constitute a relevant contribution to the description of the MtInhA flexibility by highlighting the importance of the quaternary structure to the description of its conformational changes.

## Supplementary information

Supplementary Ionformation
