# Supplementary material for: The importance of the quaternary structure to represent conformational ensembles of the major Mycobacterium tuberculosis drug target
Source: Sci Rep. 2019 Sep 23;9:13683. doi: 10.1038/s41598-019-50213-0 (PMC6757107; doi:10.1038/s41598-019-50213-0)
Supplement: Supplementary file 1 — Supplementary Ionformation [file 41598_2019_50213_MOESM1_ESM.pdf]

SUPPLEMENTARY INFORMATION:

The importance of the quaternary structure to represent conformational ensembles of the major *Mycobacterium tuberculosis* drug target

Renata Fioravanti Tarabini[a,b], Luís Fernando Saraiva Macedo Timmers[a,b,*], Carlos Eduardo Sequeiros-Borja[a,b] and Osmar Norberto de Souza[a,b*].

[a]Laboratório de Bioinformática, Modelagem e Simulação de Biossistemas (LABIO), Pontifícia Universidade Católica do Rio Grande do Sul (PUCRS), Av. Ipiranga 6681, 90619-900, Porto Alegre, RS, Brazil.

[b]Programa de Pós-Graduação em Biologia Celular e Molecular, PUCRS, Porto Alegre, RS, Brazil.

Present address:

Dr. Luis Fernando S.M. Timmers, Programa de Pós-Graduação em Biotecnologia (PPGBiotec), Universidade do Vale do Taquari -Univates, Rua Avelino Talini, 171 - Bairro Universitário, Lajeado, RS, Brasil.

M.Sc. Carlos Eduardo Sequeiros-Borja, Faculty of Biology, Institute of Molecular Biology and Biotechnology, Department of Gene Expression, Laboratory of Biomolecular Interactions and Transport, Adam Mickiewicz University in Poznań.

***Corresponding authors:** Dr. Osmar Norberto de Souza
e-mail**:** **osmar.norberto@pucrs.br**
Dr. Luis Fernando S.M. Timmers
e-mail **luis.timmers@univates.br**

Contents:

Figure SF1. Monomeric and tetrameric forms used in the molecular dynamics simulations studies. (A) MtInhA monomeric form and (B) MtInhA tetrameric form.

Figure SF2. Probability density plots of distance of SB-loop and A-loop by the distance of A-loop and B-loop. (A) MtInhA apo form, (B) MtInhA:NADH, (C) MtInhA:NADH:THT monomeric ensembles. (D) MtInhA apo form, (E) MtInhA:NADH , and (F) MtInhA:NADH:THT tetrameric ensembles.

Figure SF3. Energetic profiles of MtInhA interface. (A) MtInhA;Apo tetrameric form, (B) MtInhA:NADH tetrameric form, and (C) MtInhA:NADH-THT tetrameric form. Each line represents an interface of the tetramer, AB (black), AC (red), AD (green), BC (blue), BD (yellow), and CD (brown).

Figure SF4. Root mean square deviation (RMSD) of backbone atoms of (A) A-, (B) B-, and (C) SBL-loops. Each line represents a specific system MtInhA:Apo (black), MtInhA:NADH (red), and MtInhA:NADH-THT (green).

Figure SF5. Root mean square deviation (RMSD) of backbone atoms of A-, B-, and SBL-loops to each subunit of the tetramer. The RMSD of A-, B-, and SBL-loops for MtInhA:Apo are represented in (A), (D), and (G), respectively. The RMSD of A-, B-, and SBL-loops for MtInhA:NADH are represented in (B), (E), and (H), respectively. The RMSD of A-, B-, and SBL-loops for MtInhA:Apo are represented in (C), (F), and (I), respectively. The

Table ST1. Contribution of the first and the sum of the first 50 eigenvectors to each system, according to the principal component analysis.

Table ST2. Statistical analysis of pincer angle distributions between monomeric and tetrameric structures.

Table ST3. Statistical analysis of area distribution between monomeric and tetrameric ensembles.

Table ST4. Statistical analysis of SB-loop and A-loop distances distributions between monomeric and tetrameric ensembles.

Table ST5. Statistical analysis of B-loop and A-loop distances distributions between monomeric and tetrameric ensembles.

Table ST6. Analyses of the protein-protein interfaces regarding A-, B-, and SBL-loops.

Figure SF1. Monomeric and tetrameric forms used in the molecular dynamics simulations studies. (A) MtInhA monomeric form and (B) MtInhA tetrameric form.

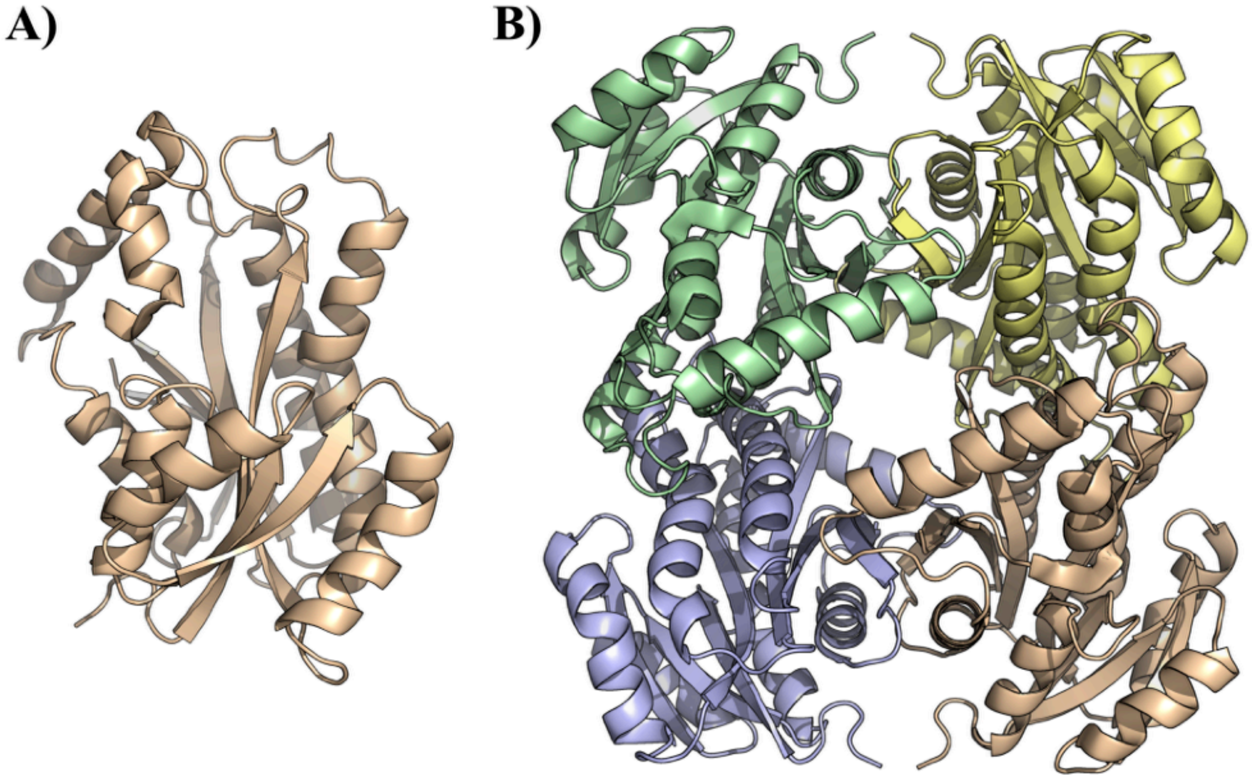

Figure SF2. Probability density plots of distance of SB-loop and A-loop by the distance of A-loop and B-loop. (A) MtInhA apo form, (B) MtInhA:NADH, (C) MtInhA:NADH:THT monomeric ensembles. (D) MtInhA apo form, (E) MtInhA:NADH , and (F) MtInhA:NADH:THT tetrameric ensembles.

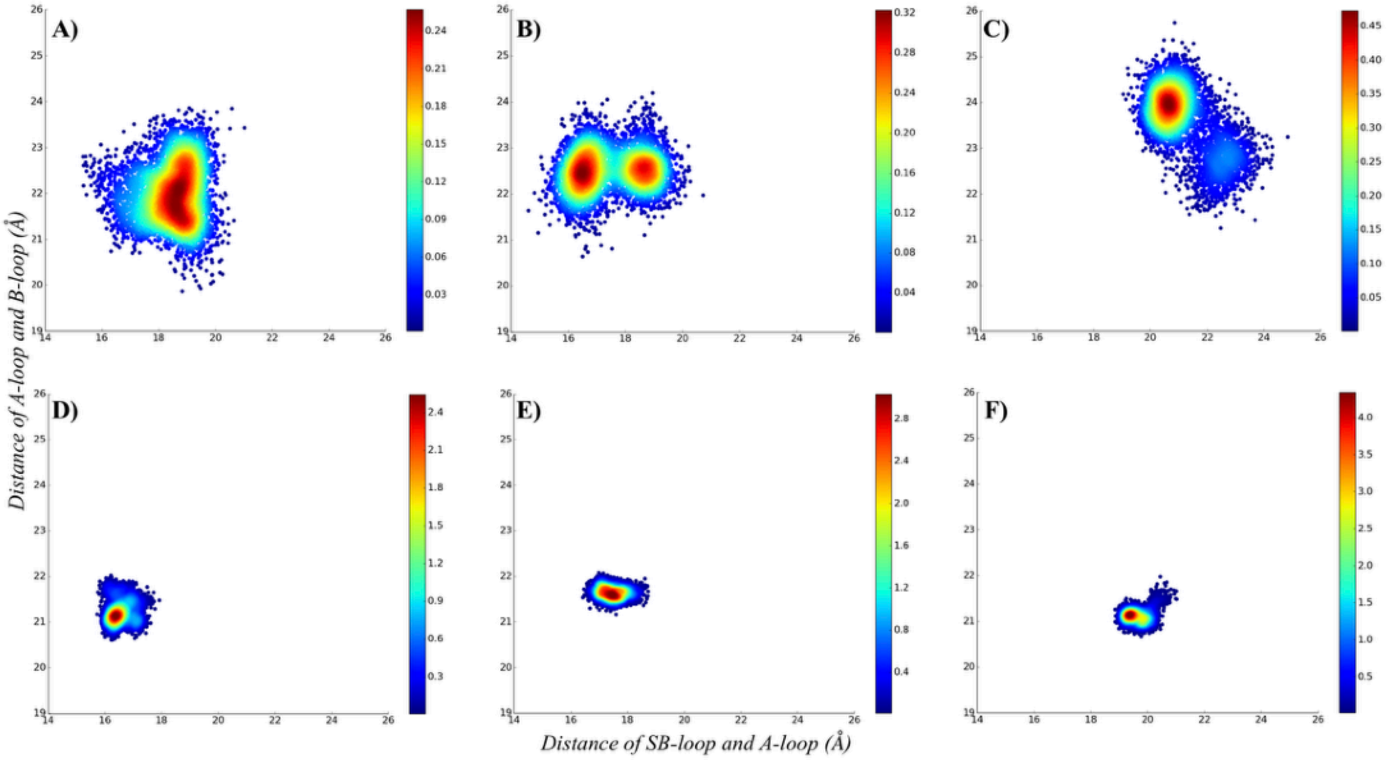

Figure SF3. Energetic profiles of MtInhA interface. (A) MtInhA;Apo tetrameric form, (B) MtInhA:NADH tetrameric form, and (C) MtInhA:NADH-THT tetrameric form. Each line represents an interface of the tetramer, AB (black), AC (red), AD (green), BC (blue), BD (yellow), and CD (brown).

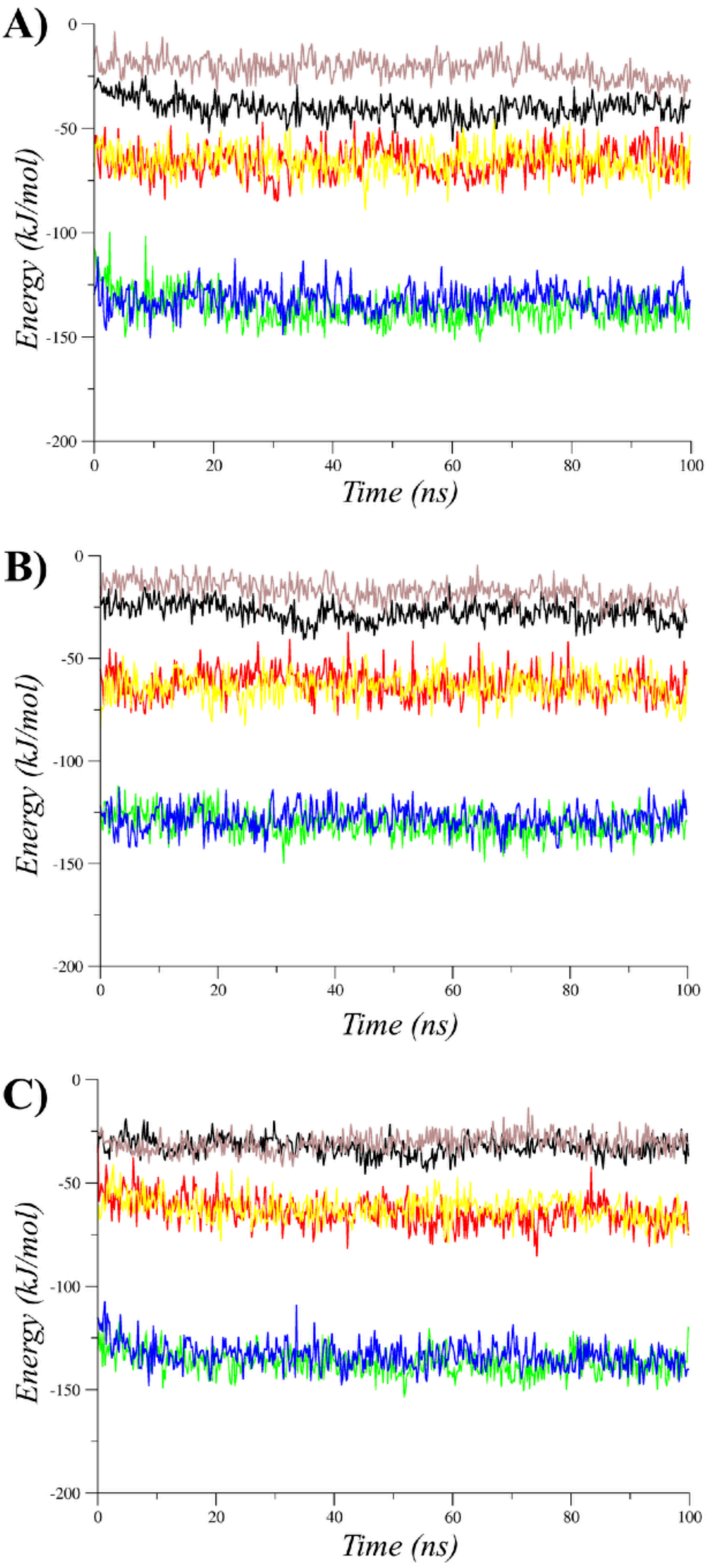

Figure SF4. Root mean square deviation (RMSD) of backbone atoms of (A) A-, (B) B-, and (C) SBL-loops. Each line represents a specific system MtInhA:Apo (black), MtInhA:NADH (red), and MtInhA:NADH-THT (green).

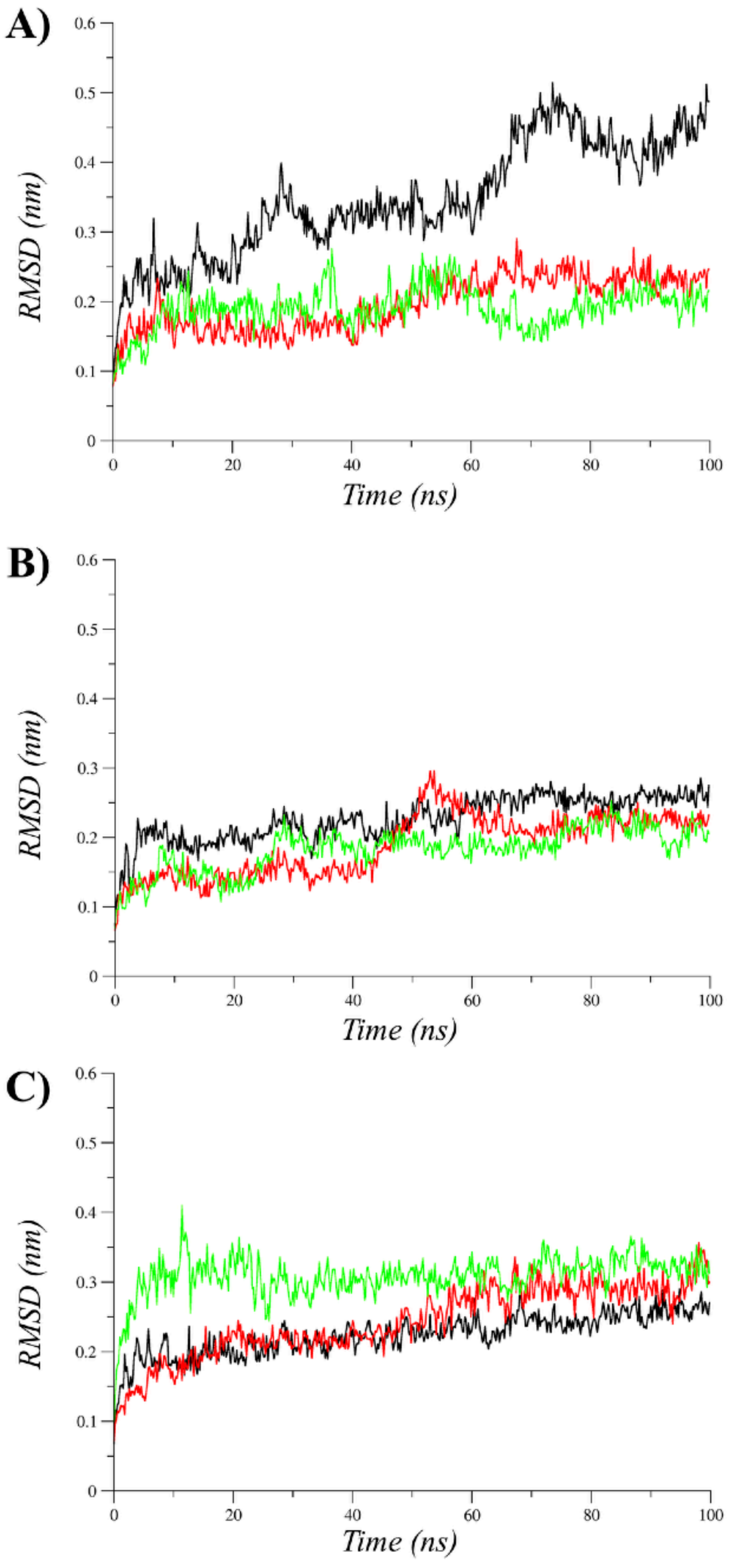

Figure SF5. Root mean square deviation (RMSD) of backbone atoms of A-, B-, and SBL-loops to each subunit of the tetramer. The RMSD of A-, B-, and SBL-loops for MtInhA:Apo are represented in (A), (D), and (G), respectively. The RMSD of A-, B-, and SBL-loops for MtInhA:NADH are represented in (B), (E), and (H), respectively. The RMSD of A-, B-, and SBL-loops for MtInhA:NADH-THT are represented in (C), (F), and (I), respectively. The subunits are represented by different line, where A, B, C, and D are colored in black, red, green, and blue, respectively.

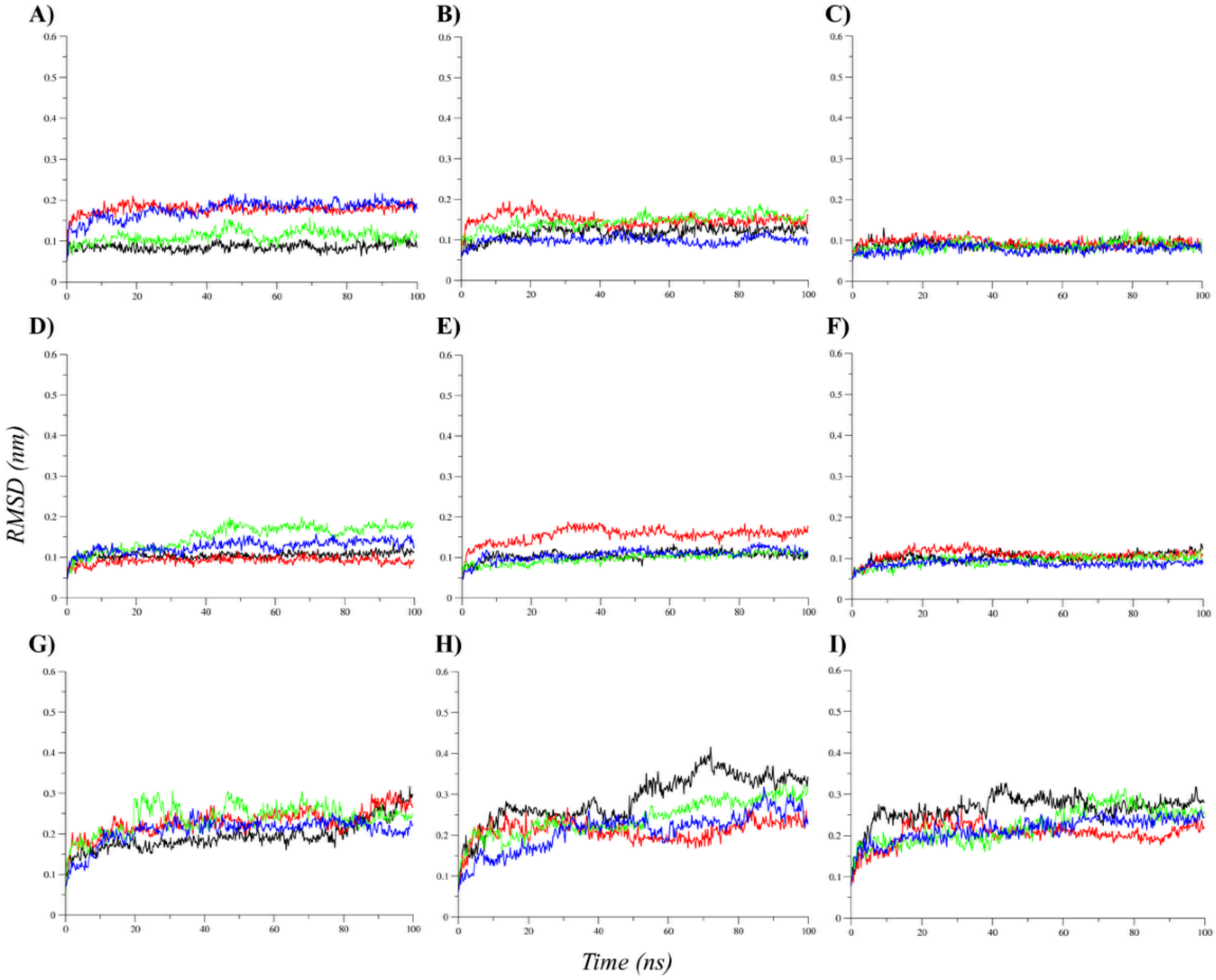

Supplementary Table ST1. Contribution of the first and the sum of the first 50

Ensemble/System	***First eigenvector (%)***			***Sum of the 50 first eigenvectors (%)***		
	Simulation 1	*Simulation 2*	*Simulation 3*	*Simulation 1*	*Simulation 2*	*Simulation 3*
Monomer:Apo	41.63	26.29	22.05	91.98	56.18	85.78
Monomer:NADH	41.52	23.78	45.96	90.31	84.68	90.33
Monomer:NADH:THT	41.13	38.49	21.86	90.62	89.14	85.33
Tetramer:Apo	22.71	24.70	20.06	73.33	76.95	77.02
Tetramer:NADH	20.55	31.95	16.78	70.84	81.78	69.34
Tetramer:NADH:THT	19.87	25.86	17.26	72.36	74.50	73.11

eigenvectors to each system, according to the principal component analysis.

Supplementary Table ST2. Statistical analysis of pincer angle distributions between monomeric and tetrameric structures.

Pincer angle

Ensembles	System	p.adjust-ed
Tetramer_A - Monomer	*Apo*	<0.01
Tetramer_B - Monomer	*Apo*	<0.01
Tetramer_C - Monomer	*Apo*	<0.01
Tetramer_D - Monomer	*Apo*	<0.01
Tetramer_A - Monomer	*NADH*	<0.01
Tetramer_B - Monomer	*NADH*	<0.01
Tetramer_C - Monomer	*NADH*	<0.01
Tetramer_D - Monomer	*NADH*	<0.01
Tetramer_A - Monomer	*NADH and substrate analogue*	<0.01
Tetramer_B - Monomer	*NADH and substrate analogue*	<0.01
Tetramer_C - Monomer	*NADH and substrate analogue*	<0.01
Tetramer_D - Monomer	*NADH and substrate analogue*	<0.01

Supplementary Table ST3. Statistical analysis of area distributions between monomeric and tetrameric ensembles.

Area

Ensembles	System	p.adjusted
Tetramer_A - Monomer	*Apo*	<0.01
Tetramer_B - Monomer	*Apo*	<0.01
Tetramer_C - Monomer	*Apo*	<0.01
Tetramer_D - Monomer	*Apo*	<0.01
Tetramer_A - Monomer	*NADH*	<0.01
Tetramer_B - Monomer	*NADH*	<0.01
Tetramer_C - Monomer	*NADH*	<0.01
Tetramer_D - Monomer	*NADH*	<0.01
Tetramer_A - Monomer	*NADH and substrate analogue*	<0.01
Tetramer_B - Monomer	*NADH and substrate analogue*	<0.01
Tetramer_C - Monomer	*NADH and substrate analogue*	<0.01
Tetramer_D - Monomer	*NADH and substrate analogue*	<0.01

Supplementary Table ST4. Statistical analysis of SB-loop and A-loop distances distributions between monomeric and tetrameric ensembles.

Distance of SB-loop and A-loop

Ensembles	System	p.adjusted
Tetramer_A - Monomer	*Apo*	<0.01
Tetramer_B - Monomer	*Apo*	<0.01
Tetramer_C - Monomer	*Apo*	<0.01
Tetramer_D - Monomer	*Apo*	<0.01
Tetramer_A - Monomer	*NADH*	<0.01
Tetramer_B - Monomer	*NADH*	<0.01
Tetramer_C - Monomer	*NADH*	<0.01
Tetramer_D - Monomer	*NADH*	<0.01
Tetramer_A - Monomer	*NADH and substrate analogue*	<0.01
Tetramer_B - Monomer	*NADH and substrate analogue*	<0.01
Tetramer_C - Monomer	*NADH and substrate analogue*	<0.01
Tetramer_D - Monomer	*NADH and substrate analogue*	<0.01

Supplementary Table ST5. Statistical analysis of B-loop and A-loop distances distributions between monomeric and tetrameric ensembles.

Distance of B-loop and A-loop

Ensembles	System	p.adjusted
Tetramer_A - Monomer	Apo	<0.01
Tetramer_B - Monomer	Apo	<0.01
Tetramer_C - Monomer	Apo	<0.01
Tetramer_D - Monomer	Apo	<0.01
Tetramer_A - Monomer	NADH	<0.01
Tetramer_B - Monomer	NADH	<0.01
Tetramer_C - Monomer	NADH	<0.01
Tetramer_D - Monomer	NADH	<0.01
Tetramer_A - Monomer	NADH and substrate analogue	<0.01
Tetramer_B - Monomer	NADH and substrate analogue	<0.01
Tetramer_C - Monomer	NADH and substrate analogue	<0.01
Tetramer_D - Monomer	NADH and substrate analogue	<0.01

Table ST6. Analyses of the protein-protein interfaces regarding A-, B-, and SBL-loops.

		APO	
Interfaces	***A-loop***	***B-loop***	***SBL-loop***
**AB**	0.00%	33.33%	11.63%
**AC**	0.00%	0.00%	6.98%
**AD**	40.00%	66.67%	1.16%
**BC**	40.00%	63.89%	1.16%
**BD**	0.00%	0.00%	6.98%
**CD**	0.00%	30.55%	10.46%

		NADH	
Interfaces	***A-loop***	***B-loop***	***SBL-loop***
**AB**	0.00%	30.55%	12.79%
**AC**	0.00%	0.00%	6.98%
**AD**	40.00%	66.67%	0.00%
**BC**	40.00%	61.11%	1.16%
**BD**	0.00%	0.00%	6.98%
**CD**	0.00%	36.11%	11.63%

		NADH:THT	
Interfaces	***A-loop***	***B-loop***	***SBL-loop***
**AB**	0.00%	33.33%	13.95%
**AC**	0.00%	0.00%	6.98%
**AD**	40.00%	63.89%	0.00%
**BC**	40.00%	61.11%	0.00%
**BD**	0.00%	0.00%	6.98%
**CD**	0.00%	30.55%	13.95%
